# NADPH oxidase-mediated redox signal contributes to lipoteichoic acid-induced MMP-9 upregulation in brain astrocytes

**DOI:** 10.1186/1742-2094-9-110

**Published:** 2012-07-06

**Authors:** Hsi-Lung Hsieh, Chih-Chung Lin, Ruey-Horng Shih, Li-Der Hsiao, Chuen-Mao Yang

**Affiliations:** 1 Department of Nursing, Division of Basic Medical Sciences, Chang Gung University of Science and Technology, Tao-Yuan, Taiwan; 2 Department of Anesthetics, Chang Gung Memorial Hospital and College of Medicine, Chang Gung University, Kwei-San, Tao-Yuan, Taiwan; 3 Department of Pharmacology and Health Aging Research Center, College of Medicine, Chang Gung University, Tao-Yuan, Taiwan; 4 Department of Pharmacology, College of Medicine, Chang Gung University, 259 Wen-Hwa 1st Road, Kwei-San, Tao-Yuan, Taiwan

## Abstract

**Background:**

Lipoteichoic acid (LTA) is a component of gram-positive bacterial cell walls and may be elevated in the cerebrospinal fluid of patients suffering from meningitis. Among matrix metalloproteinases (MMPs), MMP-9 has been observed in patients with brain inflammatory diseases and may contribute to the pathology of brain diseases. Moreover, several studies have suggested that increased oxidative stress is implicated in the pathogenesis of brain inflammation and injury. However, the molecular mechanisms underlying LTA-induced redox signal and MMP-9 expression in brain astrocytes remain unclear.

**Objective:**

Herein we explored whether LTA-induced MMP-9 expression was mediated through redox signals in rat brain astrocytes (RBA-1 cells).

**Methods:**

Upregulation of MMP-9 by LTA was evaluated by zymographic and RT-PCR analyses. Next, the MMP-9 regulatory pathways were investigated by pretreatment with pharmacological inhibitors or transfection with small interfering RNAs (siRNAs), Western blotting, and chromatin immunoprecipitation (ChIP)-PCR and promoter activity reporter assays. Moreover, we determined the cell functional changes by migration assay.

**Results:**

These results showed that LTA induced MMP-9 expression via a PKC(α)-dependent pathway. We further demonstrated that PKCα stimulated p47^phox^/NADPH oxidase 2 (Nox2)-dependent reactive oxygen species (ROS) generation and then activated the ATF2/AP-1 signals. The activated-ATF2 bound to the AP-1-binding site of MMP-9 promoter, and thereby turned on MMP-9 gene transcription. Additionally, the co-activator p300 also contributed to these responses. Functionally, LTA-induced MMP-9 expression enhanced astrocytic migration.

**Conclusion:**

These results demonstrated that in RBA-1 cells, activation of ATF2/AP-1 by the PKC(α)-mediated Nox(2)/ROS signals is essential for upregulation of MMP-9 and cell migration enhanced by LTA.

## Background

Matrix metalloproteinases (MMPs) comprise a family of calcium- and zinc-dependent proteinases, and are involved in normal development and wound healing as well as in pathological conditions such as atherosclerosis and metastasis. In brain, MMP-9 has been shown to be upregulated during various CNS diseases [1,2]. Previous reports have indicated that a series of functional element-binding sites have been identified, including NF-κB, Ets and AP-1 within the MMP-9 promoter [3], which can be regulated by diverse stimuli. Moreover, proinflammatory factors including cytokines, endotoxins and oxidative stress have been reported to upregulate MMP-9 in astrocytes in vitro [4-6], implying that MMP-9 activity may be regulated by diverse factors in the CNS during neuroinflammation.

It is worth noting that bacterial infections have been found to trigger brain inflammatory diseases [7]. Gram-positive bacterial infections of the CNS occur in bacterial meningitis and brain abscess, being localized to the membranes surrounding the brain or in its parenchyma, respectively [8]. In the CNS, the glial cells such as astrocytes and microglia are regarded as targets in gram-positive bacterial infection [9,10]. Lipoteichoic acid (LTA) is a major component of gram-positive bacterial cell walls that induces glial inflammatory activation in vitro and in vivo [11], mediated through TLR2 signaling [12]. In astrocytes, TLR signaling has been shown to be involved in brain inflammatory responses [13], accompanied by upregulation of several genes with proinflammatory and proapoptotic capabilities [14]. However, the role of MMP-9 in astrocytes, the major regulator of fundamental biological functions of the CNS [15], in LTA-induced brain inflammation remains poorly defined.

TLR2 is believed to be responsible for LTA recognition challenged by gram-positive bacteria such as *Staphylococcus aureus* and *Streptococcus pneumouniae*[16]. Upon binding to TLR heterodimers (i.e., TLR2/TLR1 or TLR2/TLR6 complex), LTA exerts a sequential activation of members of IL-1 receptor-associated kinase (IRAK) family and tumor necrosis factor receptor-associated factor 6 (TRAF6), mediated by a TLR adaptor protein, MyD88. Ultimately, TLR signaling activates the MAPK family, NF-κB and AP-1, leading to expression of cytokines and other proinflammatory proteins [7,8]. Our previous studies have demonstrated that interleukin-1 (IL-1β), bradykinin (BK) and oxidized low-density lipoprotein (oxLDL) upregulate MMP-9 expression via NF-κB, Elk-1 and AP-1 signaling in rat astrocytes [4,17,18]. In response to pathogenic ligands, TLR2/MyD88 activates PI3K/Akt, MAPKs and NF-κB pathways, which modulate immune responses following ligand recognition [16,19]. Moreover, activation of these signaling cascades and transcription factors has been reported to be involved in induction of MMP-9 in rat astrocytes [4,17,18]. A recent report has shown that LTA increases MMP-9 expression via the ERK pathway in RAW 264.7 macrophages [20]. However, the mechanisms underlying the regulation of MMP-9 expression by LTA in astrocytes are still unclear.

Reactive oxygen species (ROS) are produced by various enzymatic reactions such as NADPH oxidase (Nox). ROS are essential for many physiological functions and killing invading microorganisms [21,22]. Under pathological conditions, increasing ROS production by external stimuli can regulate the expression of several inflammatory genes during brain injury [23-25]. Recently, increasing evidence has attributed the cellular damage in neurodegenerative disorders such as AD to oxidative stress, which leads to the generation of free radicals, which are responsible for brain inflammatory disorders [24,25]. Although the effects of LTA on ROS generation have been reported in tracheal smooth muscle cells and renal diseases [26,27], LTA-induced astrocytic responses through the ROS signal are not well characterized.

Here we investigate the molecular mechanisms underlying LTA-induced MMP-9 expression in cultured RBA-1 cells. These findings demonstrate that in RBA-1 cells, LTA-induced MMP-9 expression is mediated through PKCα and Nox2-dependent generation of the ROS signaling pathway, and ATF2/AP-1. Moreover, upregulation of MMP-9 expression is positively associated with cell migration in these cells.

## Methods

### Materials

DMEM/F-12 medium, FBS, TRIzol and siRNAs of PKCα, p47^phox^, Nox1, Nox2, Nox4, ATF2 and p300 were from Invitrogen (Carlsbad, CA). Hybond C membrane and the ECL Western blotting detection system were from GE Healthcare Biosciences (Buckinghamshire, UK). MMP-9 antibody was from NeoMarker (Fremont, CA). PKCα and phospho-ATF2 antibodies were from Cell Signaling (Danver, MA). p47^phox^, Nox2 and phospho-p300 antibodies were from Santa Cruz (Santa Cruz, CA). GAPDH antibody was from Biogenesis (Boumemouth, UK). GF109203X, Gö6976, apocynin, diphenylene iodonium (DPI), tanshinone IIA (TSIIA) and GR343 were from Biomol (Plymouth Meeting, PA). Bicinchoninic acid (BCA) protein assay reagent was from Pierce (Rockford, IL). LTA (from *Staphylococcus aureus*), N-acetyl cysteine (NAC), enzymes and other chemicals were from Sigma (St. Louis, MO).

### Cell cultures

The rat brain astrocytic cell line (RBA-1) originated from a primary astrocyte culture of neonatal rat cerebrum, and was naturally developed through successive cell passages [28] and used throughout this study. Primary astrocyte cultures were prepared from the cortex of 6-day-old Sprague–Dawley rat pups [29]. Rats were maintained according to the Guidelines of the Animal Care Committee of Chang Gung University and the National Institutes of Health Guide for the Care and Use of Laboratory Animals. The purity of primary astrocyte cultures was assessed using an astrocyte-specific marker, GFAP, showing over 95% GFAP-positive astrocytes. Cells were plated onto 12-well culture plates and made quiescent at confluence by incubation in serum-free DMEM/F-12 for 24 h. Growth-arrested cells were incubated with LTA at 37°C for the indicated time intervals. When the inhibitors were used, cells were pretreated with the inhibitor for 1 h before exposure to LTA.

### MMP gelatin zymography

Growth-arrested cells were incubated with LTA for the indicated time intervals. Treatment of RBA-1 cells with pharmacological inhibitors or LTA alone had no significant effect on cell viability determined by an XTT assay (data not shown). The cultured media were analyzed by gelatin zymography [29]. Gelatinolytic activity was manifested as horizontal white bands on a blue background. Because cleaved MMPs were not reliably detectable, only pro-form zymogens were quantified.

### Total RNA extraction and reverse transcription PCR analysis

Total RNA was extracted from RBA-1 cells [29]. The cDNA obtained from 0.5 μg total RNA was used as a template for PCR amplification. Oligonucleotide primers were designed based on Genbank entries for rat MMP-9, Nox1-4 [30] and β-actin. The following primers were used for amplification reaction: MMP-9, 5′-AGTTTGGTGTCGCGGAGCAC-3′ (sense), 5′-TACATGAGCGCTTCCGGCAC-3′ (antisense); Nox1, 5′-TACGAAGTGGCTGTACTGGTTG-3′ (sense), 5′-CTCCCAAAGGAGGTTTTCTGTT-3′ (antisense); Nox2, 5′-TCAAGTGTCCCCAGGTATCC-3′ (sense), 5′-CTTCACTGGCTGTACCAAAGG-3′ (antisense); Nox3, 5′-AATCACAGAGTCTGCCTGGACT-3′ (sense), 5′-ATCCAGACTTTCATCCCAGTGT-3′ (antisense); Nox4, 5′-GGAAGTCCATTTGAGGAGTCAC-3′ (sense), 5′-TGGATGTTCACAAAGTCAGGTC-3′ (antisense); β-actin, 5′-GAACCCTAAGGCCAACCGTG-3′ (sense) and 5′-TGGCATAGAGGTCTTTACGG-3′ (antisense). The amplification was performed in 30 cycles at 55°C, 30 s; 72°C, 1 min; 94°C, 30s. PCR fragments were analyzed on 2% agarose in 1X TAE gel containing ethidium bromide, and their size was compared to molecular weight markers. Amplification of β-actin, a relatively invariant internal reference RNA, was performed in parallel, and cDNA amounts were standardized to equivalent β-actin mRNA levels. These primer sets specifically recognize only the genes of interest as indicated by amplification of a single band of the expected size (754 bp for MMP-9, 337 bp for Nox1, 209 bp for Nox2, 214 bp for Nox3, 244 bp for Nox4 and 514 bp for β-actin) and direct sequence analysis of the PCR product. Real-time PCR was performed with the TaqMan gene expression assay system, using primers and probe mixes for MMP-9 and endogenous GAPDH control genes. PCRs were performed using the 7500 Real-Time PCR System (Applied Biosystems, Foster City, CA). The primers were: 5′-(TGATGCCATTGCTGATATCCA)-3′ (sense), 5′-(CGGATCCTCAAAGGCTGAGT)-3′ (anti-sense) for MMP-9; 5′-(AACTTTGGCATCGTGGAAGG)-3′ (sense), 5′-(GTGGATGCAGGGATGATGTTC)-3′ (anti-sense) for GAPDH. Relative gene expression was determined by the ΔΔCt method, where Ct meant threshold cycle. All experiments were performed in triplicate (*n* = 3).

### Preparation of cell extracts and western blotting analysis

Growth-arrested cells were incubated with LTA for the indicated time intervals. The cell lysates were collected and the protein concentration was determined by the BCA reagents. Samples from these cell lysates (30 μg protein) were denatured and subjected to SDS-PAGE using a 10% (w/v) running gel. The phosphorylation of serine, ATF2, and p300 or total Nox, p47^phox^, PKCα, ATF2 and p300 were identified and quantified by Western blotting as previously described [29]. The immunoreactive bands were detected by the UVP Biospectrum® imaging system (Upland, CA).

### Measurement of intracellular ROS generation

The peroxide-sensitive fluorescent probe 2′,7′-dichlorofluorescein diacetate (DCF-DA) was used to assess the generation of intracellular ROS [31] with minor modifications. RBA-1 cells in monolayers were incubated with RPMI-1640 supplemented with 5 μM DCF-DA for 45 min at 37°C. The supernatant was replaced with fresh RPMI-1640 medium before stimulation with LTA (50 μg/ml). Relative fluorescence intensity was recorded over time (0–60 min) by using a fluorescent plate reader (Thermo, Appliskan) at an excitation wavelength of 485 nm, and emission was measured at a wavelength of 530 nm. Fluorescent images were also obtained by using fluorescence microscopy (ZEISS, Axiovert 200 M). The data presented are generated from three separate assays (*n* = 3).

### Determination of NADPH oxidase activity by chemiluminescence assay

The NADPH oxidase activity in intact cells was assayed by lucigenin chemiluminescence assay as described previously [32] with modifications. After incubation, the cells were gently scraped and centrifuged at 400 × g for 10 min at 4°C. The cell pellet was resuspended in a volume (35 μl/well) of ice-cold RPMI 1640 medium, and the cell suspension was kept on ice. To a final 200 μl volume of pre-warmed (37°C) RPMI 1640 medium containing either NADPH (1 μM) or lucigenin (20 μM), 5 μl of cell suspension (2 × 10^4^ cells) was added to initiate the reaction followed by immediate measurement of chemiluminescence in a Appliskan luminometer (Thermo®) in out-of-coincidence mode. Appropriate blanks and controls were established, and chemiluminescence was recorded. Neither NADPH nor NADH enhanced the background chemiluminescence of lucigenin alone (30–40 counts/min). Chemiluminescence was continuously measured for 12 min, and the activity of NADPH oxidase was expressed as counts per million cells.

### Isolation of cell fraction

After treatment, cells were harvested, sonicated for 5 s at output 1.5 with a sonicator (Misonix, Inc., Farmingdale, NY) and centrifuged at 8,000 rpm for 15 min at 4°C. The pellet was collected as the nuclear fraction. The supernatant was centrifuged at 14,000 rpm for 60 min at 4°C to yield the pellet (membrane fraction) and the supernatant (cytosolic fraction).

### Immunofluorescence staining

Growth-arrested cells were treated with LTA (50 μg/ml), washed twice with ice-cold PBS, fixed with 4% (w/v) paraformaldehyde in PBS for 30 min and then permeabilized with 0.3% Triton X-100 in PBS for 15 min. The staining was performed by incubating with 10% normal goat serum in PBS for 30 min followed by incubating with an anti-p47^phox^ polyclonal antibody (1:200 dilution) for 1 h in PBS with 1% BSA, washing three times with PBS, incubating for 1 h with fluorescein isothiocyanate (FITC)-conjugated goat anti-rabbit antibody (1:200 dilution) in PBS with 1% BSA, washing three times with PBS and finally mounting with aqueous mounting medium. The images were observed under a fluorescence microscope (ZEISS, Axiovert 200 M). The data presented are generated from three separate assays (*n* = 3).

### Transient transfection with siRNAs

The small interfering RNA (siRNA) duplexes corresponding to rat PKCα, p47^phox^, Nox1-4, ATF2, p300 and scrambled siRNA were from Invitrogen (Invitrogen, Carlsbad, CA). Transient transfection of siRNAs (100 nM) was carried out using Lipofetamine^TM^ RNAiMAX reagent according to the manufacturer’s instructions (*n* = 3).

### Plasmid construction, transient transfection and promoter activity assay

The upstream region (−1280 to +19) of the rat MMP-9 promoter was cloned to the pGL3-basic vector containing the luciferase reporter system. Briefly, a 1.3-kb segment at the 5′-flanking region of the rat MMP-9 gene was amplified by PCR using specific primers from the rat MMP-9 gene (accession no. U36476): 5′-ccccggtaccGAAGGCGAAATGCTTTGCCC (forward/Kpn1) and 5′-ccccctcgaGGGTGAGAACCGAAGCTTCTG (reverse/Xho1). The pGL3-Basic vector, containing a polyadenylation signal upstream from the luciferase gene, was used to construct the expression vectors by subcloning PCR-amplified DNA of the MMP-9 promoter into the Kpn1/Xho1 site of this vector. The PCR products (pGL3-MMP-9WT) were confirmed by their size, as determined by electrophoresis, and by DNA sequencing. All plasmids were prepared by using QIAGEN plasmid DNA preparation kits. The MMP-9-luc constructs were transfected into RBA-1 cells, respectively, using a Lipofectamine reagent according to the manufacturer’s instructions. The transfection efficiency (~60%) was determined by transfection with enhanced GFP. After incubation with LTA (50 ng/ml), cells were collected and disrupted by sonication in lysis buffer (25 mM Tris, pH 7.8, 2 mM EDTA, 1% Triton X-100 and 10% glycerol). After centrifugation, aliquots of the supernatants were tested for luciferase activity using the luciferase assay system. Firefly luciferase activities were standardized for β-galactosidase activity. The data presented are generated from three separate assays (*n* = 3).

### Chromatin immunoprecipitation assay

To detect the in vivo association of nuclear proteins with rat *mmp-9* promoter, chromatin immunoprecipitation (ChIP) analysis was conducted as described previously [17]. RBA-1 cells in 100-mm dishes were grown to confluence and serum starved for 24 h. After treatment with LTA, protein-DNA complexes were fixed by 1% formaldehyde in PBS. The fixed cells were washed and lysed in SDS-lysis buffer (1% SDS, 5 mM EDTA, 1 mM PMSF, 50 mM Tris–HCl, pH 8.1) and sonicated on ice until the DNA size became 200–1,000 base pairs. The samples were centrifuged, and the soluble chromatin was pre-cleared by incubation with sheared salmon sperm DNA-protein agarose A slurry (Upstate) for 30 min at 4°C with rotation. After centrifugation at 800 rpm for 1 min, one portion of the pre-cleared supernatant was used as DNA input control, and the remains were subdivided into aliquots and then incubated with a non-immune rabbit immunoglobulin G (IgG; Santa Cruz), anti-ATF2 (Santa Cruz), respectively, for overnight at 4°C. The immunoprecipitated complexes of Ab-protein-DNA were collected by using the above protein A beads and washed successively with low-salt buffer (0.1% SDS, 1% Triton X-100, 2 mM EDTA, 20 mM Tris–HCl, pH 8.1, 150 mM NaCl), high-salt buffer (same as the low-salt buffer but with 500 mM NaCl), LiCl buffer (0.25 M LiCl, 1% NP-40, 1% deoxycholate, 1 mM EDTA, 10 mM Tris–HCl, pH 8.1) and Tris-EDTA (pH 8.0), and then eluted with elution buffer (1% SDS, 100 mM NaHCO_3_). The cross-linking of protein-DNA complexes was reversed by incubation with 5 M NaCl at 65°C for 4 h, and DNA was digested with 10 μg of proteinase K (Sigma)/ml for 1 h at 45°C. The DNA was then extracted with phenol-chloroform, and the purified DNA pellet was precipitated with isopropanol. After washing, the DNA pellet was resuspended in H_2_O and subjected to PCR amplification with the forward (5′-AGAGCCTGCTCCCAGAGGGC-3′) and reverse (5′-GCCAAGTCAGGCAGGACCCC-3′), which were specifically designed from the distal AP-1 *mmp-9* promoter region (−557 to −247). PCR products were analyzed on ethidium bromide-stained agarose gels (*n* = 3).

### Migration assay

RBA-1 cells were cultured to confluence in 10-cm dishes and starved with serum-free DMEM/F-12 medium for 24 h. The monolayer cells were scratched manually with a blade to create extended and definite scratches in the center of the dishes with a bright and clear field. The detached cells were removed by washing the cells once with PBS. Serum-free DMEM/F-12 medium with or without LTA (50 μg/ml) was added to each dish as indicated after pretreatment with the inhibitors for 1 h, containing a DNA synthesis inhibitor hydroxyurea (10 μM) in the whole course. Images of migratory cells from the scratch boundary were observed and acquired at 0 and 48 h with a digital camera and a light microscope (Olympus, Japan). The number of migratory cells was counted from the resulting four phase images for each point and then averaged for each experimental condition. The data presented are generated from three separate assays.

### Statistical analysis of data

Data were estimated using a GraphPad Prism Program (GraphPad, San Diego, CA, USA). Quantitative data were analyzed using ANOVA followed by Tukey’s honestly significant difference tests between individual groups. Data were expressed as mean ± SEM of three independent experiments (*n* = 3). A value of *P* < 0.05 was considered significant.

## Results

### ROS signal is essential for LTA-induced MMP-9 expression in RBA-1 cells

**Figure 1:**
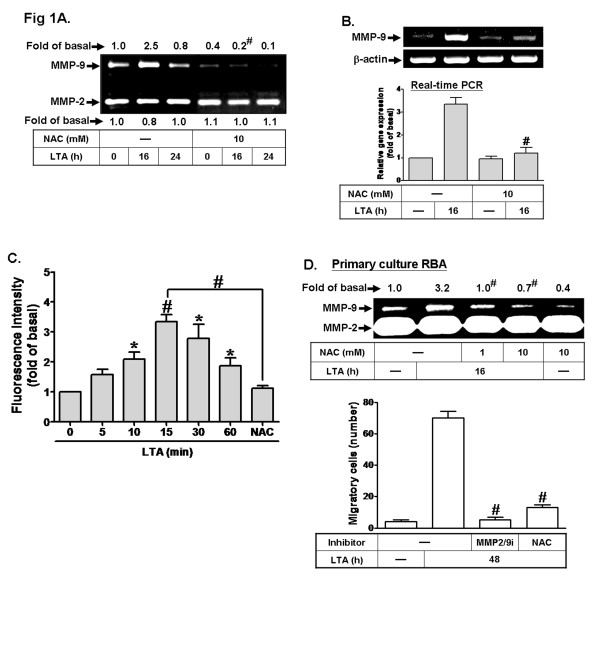
**ROS are essential for LTA-induced MMP-9 expression in RBA-1 cells. a** Cells were pretreated with or without N-acetylcysteine (NAC, 10 mM) for 1 h before exposure to 50 μg/ml LTA for the indicated time intervals. The conditioned media were analyzed by gelatin zymography for MMP-9 expression and activity. **b** Cells were treated with or without NAC (10 mM) for 1 h before exposure to 50 μg/ml LTA for 16 h. The total RNA was analyzed by RT-PCR (*upper panel*) and real-time RT-PCR (*lower panel*). **c** Cells were incubated with the peroxide-sensitive fluorescent probe DCF-DA (5 μM) for 45 min, followed by stimulation with 50 μg/ml LTA for the indicated time intervals or for 15 min in presence or absence of 10 mM NAC. The fluorescence intensity of cells was determined. **d** Rat primary astrocytes were isolated and cultured, and pretreated with or without NAC (1 or 10 mM) before exposure to LTA for 16 h. The conditioned media were analyzed by gelatin zymography (*upper panel*). For cell migration, cells were plated on coverslips and grown to confluence, then the coverslips were transferred to a new 10-cm dish containing serum-free medium for 24 h. Cells were pretreated with MMP2/9 inhibitor (2/9i) or NAC for 1 h and then incubated with LTA (50 μg/ml) for 48 h. Phase contrast images of cells were taken. The number of LTA-induced cell migrations was counted (*lower panel*). Data are expressed as the mean ± SEM of three independent experiments. ^*^P < 0.05; ^#^*P* < 0.01, as compared with the respective values of cells stimulated with vehicle (**c**) or LTA alone (**c**, **d**). The figure represents one of three similar experiments.

ROS have been shown to induce MMPs expression in various cell types [33]. To determine whether ROS participated in MMP-9 induction, pretreatment of RBA-1 cells with N-acetylcysteine (NAC, a ROS scavenger, 10 mM) attenuated the LTA-induced MMP-9 protein and mRNA expression (Figure 1a and b), suggesting that ROS may play a crucial role in LTA-induced MMP-9 expression in RBA-1 cells. To determine whether LTA stimulates intracellular ROS generation, as shown in Figure 1c, LTA stimulated ROS generation in a time-dependent manner that was markedly attenuated by pretreatment with NAC, indicating that LTA stimulated ROS generation. To demonstrate whether LTA-induced MMP-9 expression via the ROS-dependent pathway also occurred in primary cultured astrocytes, as shown in Figure 1d, pretreatment with NAC concentration-dependently inhibited LTA-induced MMP-9 expression in primary cultured astrocytes. To demonstrate the functional activity of MMP-9 induced by LTA, we evaluated cell migration of primary culture astrocytes. The images of cell migration were taken and counted at 48 h induction by LTA (50 μg/ml). As shown in Figure 1d (lower panel), LTA-induced cell migration was significantly blocked by pretreatment with the inhibitors of MMP2/9 (2/9i, 1 μM) or ROS (NAC, 10 mM). These data suggested that upregulation of MMP-9 and its activity via the ROS-dependent pathway is required for enhancing cell migration induced by LTA in brain astrocytes. Taken together, these results demonstrated that LTA-induced responses in rat primary cultured astrocytes are similar to those of RBA-1 cells. Thus, the following experiments were performed using RBA-1 cells, which can be applied as a model throughout this study.

### Nox2-derived ROS generation is involved in LTA-induced MMP-9 expression

**Figure 2:**
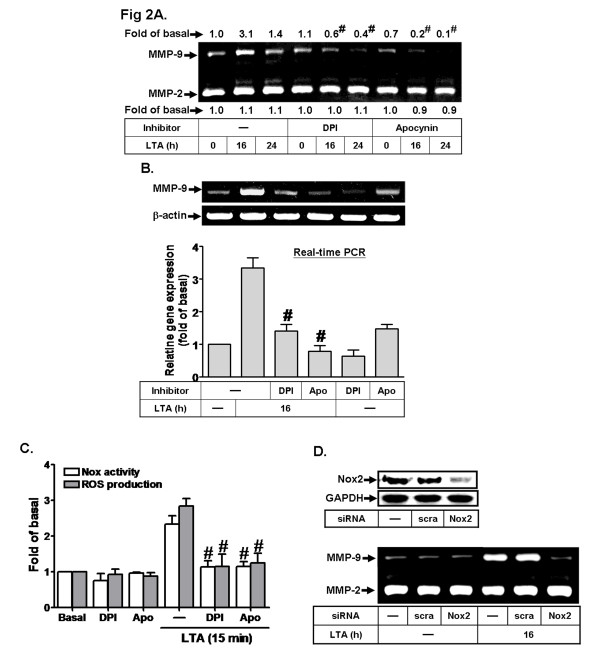
**NADPH oxidase (Nox)-dependent ROS generation is involved in LTA-induced MMP-9 expression in RBA-1 cells. a** Cells were pretreated with or without DPI (1 μM) or apocynin (Apo, 10 μM) for 1 h before exposure to LTA (50 μg/ml) for the indicated time intervals. The conditioned media were analyzed by gelatin zymography for MMP-9 expression and activity. **b** The total RNA was collected and MMP-9 mRNA expression analyzed by RT-PCR (*upper panel*) and real-time RT-PCR (*lower panel*). **c** Cells were pretreated with or without DPI (1 μM) or apocynin (Apo, 10 μM) for 1 h before exposure to 50 μg/ml LTA for 15 min. The Nox activity and ROS generation were analyzed. **d** Cells were transfected with scramble (*scra*) or Nox2 siRNA for 24 h, followed by stimulation with LTA for 16 h. The conditioned media and cell lysates were collected for gelatin zymography or Western blotting analysis of Nox2 and GAPDH (as an internal control). Data are expressed as the mean ± SEM of three independent experiments. ^*^P < 0.05; ^#^*P* < 0.01, as compared with the r cells stimulated with LTA (**c**) alone. The figure represents one of three similar experiments.

NADPH oxidase (Nox) is considered to be a major source of ROS in several physiological and pathological processes [21,34]. To further explore whether LTA-induced MMP-9 expression mediated through activation of Nox activation, as shown in Figure 2a and b, pretreatment with a Nox activity inhibitor diphenyleneiodonium (DPI, 1 μM) or p47^phox^ inhibitor apocynin (Apo, 10 μM) significantly attenuated the LTA-induced MMP-9 protein and mRNA expression in RBA-1 cells. To further demonstrate whether LTA stimulates p47^phox^-mediated Nox activity, as shown in Figure 2c, LTA stimulated Nox activity and ROS generation, which was attenuated by pretreatment with DPI (1 μM) or Apo (10 μM), suggesting that p47^phox^/Nox-derived ROS is involved in LTA-induced MMP-9 expression in RBA-1 cells. To determine which Nox isotypes are involved in these responses, the expression of Nox isotypes was analyzed, and transfection with Nox siRNA was performed. First, we found that Nox1, Nox2 and Nox4 were expressed in RBA-1 cells and Nox2 was predominantly expressed in comparison to the other two determined by RT-PCR (data not shown). Next, transfection with Nox2 siRNA knocked down the expression of Nox2 protein and significantly attenuated LTA-induced MMP-9 expression in RBA-1 cells (Figure 2d). These results suggested that LTA-induced MMP-9 expression is mediated through Nox2-dependent ROS generation in these cells.

### Involvement of p47^phox^ in LTA-induced MMP-9 expression

**Figure 3:**
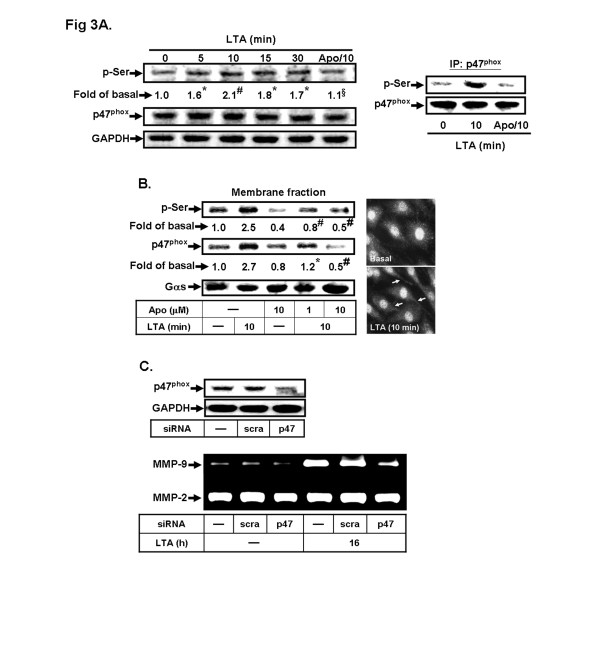
**Involvement of p47**^**phox**^**translocation in LTA-induced MMP-9 expression.** Cells were pretreated without or with Apo (10 μM) for 1 h and then stimulated with 50 μg/ml LTA for the indicated time intervals or 10 min. **a** The whole cell lysates and (**b**) the membrane fraction were prepared and analyzed by Western blotting using an anti-phospho-serine (p-Ser), p47^phox^ or Gαs (as a membrane control) antibody. The p47^phox^ translocation was also observed by immunofluorescent staining. **c** Cells were transfected with scramble (*scra*) or p47^phox^ siRNA for 24 h, followed by stimulation with LTA for 16 h. The conditioned media and cell lysates were analyzed by gelatin zymography or Western blotting analysis of p47^phox^ and GAPDH. Data are expressed as the mean of three independent experiments. ^*^P < 0.05; ^#^*P* < 0.01, as compared with the cells stimulated with vehicle (**a**) and LTA (**a**, **b**) alone. ^§^*P* < 0.01, as compared with the cells stimulated with LTA (**a**) alone. The figure represents one of three similar experiments.

Moreover, phosphorylation and translocation of p47^phox^ to plasma membrane are important events in the activation of Nox [35,36]. Therefore, we first determined whether LTA stimulates p47^phox^ phosphorylation at serine residues; as shown in Figure 3a, LTA stimulated p47^phox^ serine phosphorylation in a time-dependent manner, which was attenuated by pretreatment with Apo (10 μM). We next determined the effect of LTA on translocation of p47^phox^; cells were treated with LTA for 10 min in the absence or presence of Apo. The membrane fraction was analyzed by Western blot using an anti-phospho-serine (p-Ser) or anti-p47^phox^ antibody. LTA stimulated a significant translocation of p47^phox^ from the cytosol to the membrane with a maximal response within 10 min, which was attenuated by pretreatment with Apo (Figure 3b, left part). The results were further supported by the data of fluorescence images obtained using fluorescent microscopy (Figure 3b, right part). Moreover, to further demonstrate the role of p47^phox^ in the MMP-9 induction by LTA, as shown in Figure 3c, transfection with p47^phox^ siRNA knocked down the expression of p47^phox^ protein and significantly attenuated LTA-induced MMP-9 expression. These results suggested that p47^phox^/Nox-dependent ROS generation plays a crucial role in LTA-induced MMP-9 expression in RBA-1 cells.

### LTA induces MMP-9 expression via PKCα-dependent pathway

**Figure 4:**
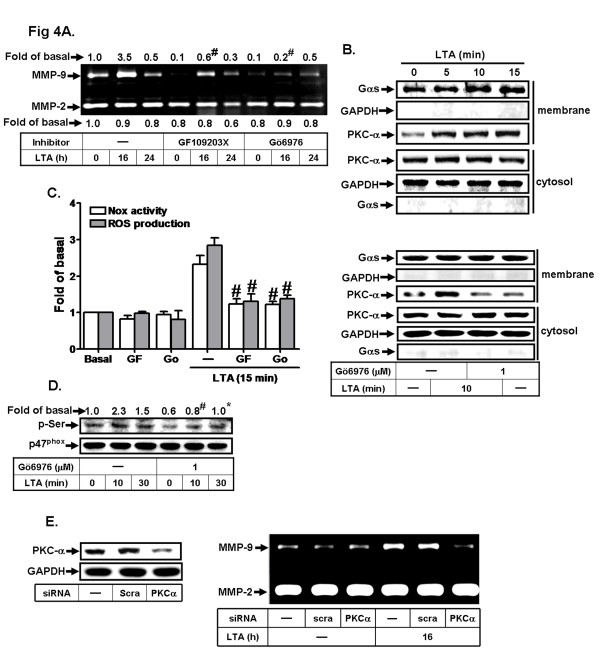
**LTA induces ROS generation and MMP-9 expression via PKCα. a** Cells were pretreated with or without GF109203X (GF, 30 μM) or Gö6976 (Gö, 1 μM) for 1 h before exposure to 50 μg/ml LTA for the indicated time intervals. The conditioned media were analyzed by gelatin zymography. **b** Cells were pretreated without or with Gö (1 μM) for 1 h and then incubated with LTA for the indicated time intervals (*upper part*) or 10 min (*lower part*). The membrane and cytosol fractions were prepared and analyzed by Western blotting using anti-PKC-α, Gαs (as a membrane control) or GAPDH antibody. **c** Cells were pretreated with or without GF or Gö for 1 h before exposure to LTA for 15 min. The Nox activity and ROS generation were analyzed. **d** Cells were pretreated with or without Gö (1 μM) for 1 h before exposure to LTA for the indicated time intervals. **e** Cells were transfected with scramble (*scra*) or PKCα siRNA for 24 h, followed by stimulation with LTA for 16 h. The conditioned media and cell lysates were analyzed by gelatin zymography or Western blotting analysis. The cell lysates were analyzed by Western blotting using anti-phospho-serine (p-Ser), p47^phox^ or GAPDH antibody. Data are expressed as the mean ± SEM of three independent experiments. ^*^P < 0.01; ^#^*P* < 0.01, as compared with the cells stimulated with LTA (**c**, **d**) alone. The figure represents one of three similar experiments.

Several PKC isoforms such as PKC-α have been shown to regulate MMP-9 expression in various cell types [37]. Recent reports also suggested that activation of Nox/ROS signaling might be mediated through PKC [36,38]. Here we further demonstrated which PKC isoforms are involved in LTA-induced Nox/ROS generation and MMP-9 expression. Pretreatment with either a pan-PKC inhibitor GF109203X (30 μM) or a selective PKCα inhibitor Gö6976 (1 μM) significantly inhibited LTA-induced MMP-9 expression (Figure 4a) as revealed by zymography, suggesting that PKC (PKC-α especially) plays a potential role in LTA-induced MMP-9 expression. LTA also stimulated PKCα translocation from the cytosol to the membrane in a time-dependent manner with a maximal increase within 10–15 min (Figure 4b, upper part), which was attenuated by pretreatment with Gö6976 (1 μM) (Figure 4b, lower part), confirming the involvement of PKCα in LTA-induced MMP-9 expression in RBA-1 cells. We further examined the role of PKC(α) in LTA-stimulated Nox activity and ROS generation; as shown in Figure 4c, LTA-stimulated Nox activation and ROS generation were blocked by pretreatment with GF109203X (GF, 30 μM) or Gö6976 (Go, 1 μM). To ensure the phosphorylation of p47^phox^ at serine residues mediated through PKC-α, as shown in Figure 4d, LTA-stimulated phosphorylation of serine residues on p47^phox^ was attenuated by pretreatment with Gö6976 (1 μM). To confirm the role of PKCα in MMP-9 induction by LTA, as shown in Figure 4e, transfection with PKCα siRNA knocked down the expression of PKCα protein and significantly attenuated LTA-induced MMP-9 expression. These results demonstrated that PKCα participates in LTA-induced Nox/ROS generation and MMP-9 expression in RBA-1 cells.

### ATF-2/AP-1 is required for LTA-induced MMP-9 expression in RBA-1 cells

**Figure 5:**
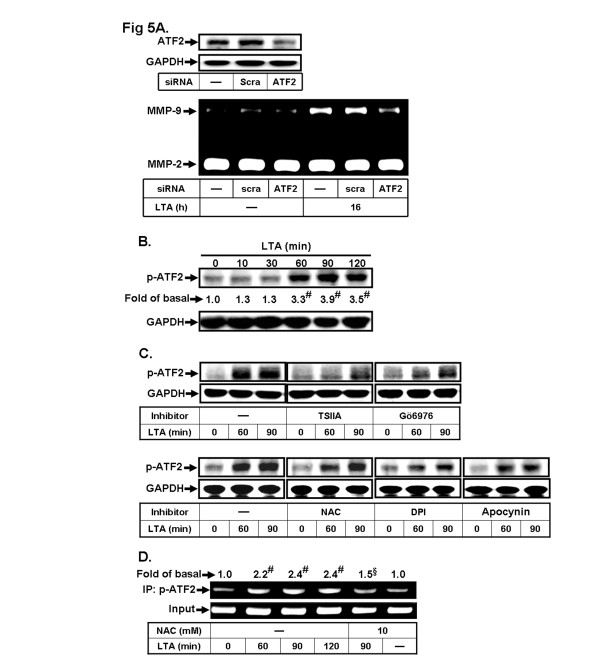
**ATF-2/AP-1 is required for LTA-induced MMP-9 expression. a** RBA-1 cells were transfected with scramble (*scra*) or ATF2 siRNA for 24 h, followed by stimulation with 50 μg/ml LTA for 16 h. The conditioned media and cell lysates were analyzed by gelatin zymography or Western blotting. **b** Cells were treated with LTA for the indicated time intervals. **c** Cells were pretreated with or without tanshinone IIA (TSIIA, 10 μM), Gö6976 (1 μM), NAC (10 mM), DPI (1 μM) or apocynin (10 μM) for 1 h before exposure to LTA for the indicated time intervals. The cell lysates were analyzed by Western blotting using an anti-phospho-ATF-2 (p-ATF2) or GAPDH antibody. **d** RBA-1 cells were pretreated with NAC (10 mM) for 1 h and then incubated with LTA (50 μg/ml) for the indicated time intervals or 90 min. The ATF-2/AP-1 binding activity was analyzed by chromatin-IP (ChIP)-PCR assay. Data are expressed as the mean of three independent experiments. ^#^*P* < 0.01, as compared with the cells stimulated with vehicle (**b**, **d**) or ^§^*P* < 0.05, as compared with the cells stimulated with LTA (**d**) alone. The figure represents one of three similar experiments.

Activating transcription factor (ATF) subfamily (ATFa, ATF-1 ATF-2 and ATF-3) is a member of AP-1 transcription factor complex. ATF-2 and Fos proteins are commonly seen in pathological processes. However, the role of ATF-2 in LTA-induced MMP-9 expression is still unknown. Thus, we investigated whether ATF2 is involved in LTA-induced MMP-9 expression in RBA-1 cells; as shown in Figure 5a, transfection with ATF-2 siRNA downregulated ATF-2 protein expression and significantly attenuated LTA-induced MMP-9 expression in RBA-1 cells, suggesting that ATF2/AP-1 is an important factor in LTA-induced MMP-9 expression. To determine whether the phosphorylation of ATF-2 is essential for LTA-induced MMP-9 expression, we found that LTA stimulated phosphorylation of ATF2 in a time-dependent manner. There was a significant increase within 60 min and reached a maximal response by 90 min (Figure 5b). Pretreatment with an AP-1 inhibitor TSIIA (10 μM) markedly attenuated LTA-stimulated ATF-2 phosphorylation (Figure 5c). To determine whether PKC and Nox/ROS are involved in LTA-stimulated ATF-2 phosphorylation, as shown in Figure 5c, pretreatment with Gö6976 (1 μM), NAC (10 mM), DPI (1 μM) or Apo (10 μM) attenuated LTA-stimulated ATF-2 phosphorylation, indicating that LTA-stimulated ATF-2/AP-1 phosphorylation was mediated through the PKCα/Nox/ROS cascade in RBA-1 cells. Here we also used the ChIP-PCR assay to determine whether ATF2/AP-1 is involved in LTA-induced MMP-9 gene expression. We first designed a pair of primers for MMP-9 promoter (−597 to −318), containing an AP-1 binding site. Chromatin was immunoprecipitated using an anti-phospho-ATF-2 antibody, and the MMP-9 promoter region (−597 to −318) was amplified by PCR. As shown in Figure 5d, in vivo binding of phospho-ATF-2 to the MMP-9 promoter was time-dependently increased by LTA, which was attenuated by NAC (Figure 5d). These results suggested that ROS-dependent signaling is involved in LTA-induced ATF-2/AP-1 binding to the MMP-9 promoter in RBA-1 cells.

### The transcriptional co-activator p300 is crucial for LTA-induced MMP-9 expression

**Figure 6:**
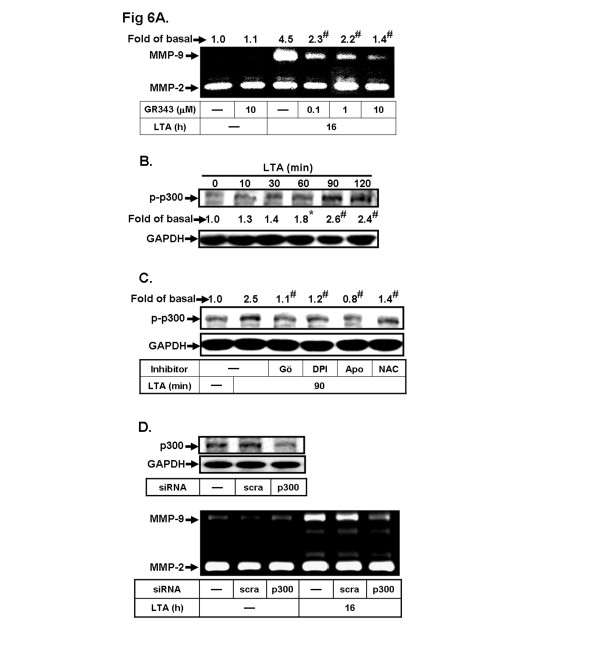
**The transcriptional co-activator p300 contributes to LTA-induced MMP-9 expression. a** Cells were pretreated with or without GR343 for 1 h before exposure to 50 μg/ml LTA for 16 h. The conditioned media were analyzed by gelatin zymography. **b**, **c** Cells were pretreated without or with Gö6976 (Gö, 1 μM), DPI (1 μM), apocynin (10 μM) or NAC (10 mM) for 1 h, and then incubated with 50 μg/ml LTA for the indicated time intervals (**b**) or 90 min (**c**). The cell lysates were analyzed by Western blotting using an anti-phospho-p300 (p-p300) or GAPDH antibody. **d** Cells were transfected with scramble (*scra*) or p300 siRNA for 24 h, followed by stimulation with 50 μg/ml LTA for 16 h. The conditioned media and cell lysates were collected and analyzed by gelatin zymography or Western blotting. Data are expressed as the mean of three independent experiments. ^*^P < 0.05; ^#^*P* < 0.01, as compared with the cells stimulated with vehicle (**b**) or LTA (**a**, **c**) alone. The figure represents one of three similar experiments.

Several studies have reported that p300, a transcriptional co-activator, plays a critical role in many gene transcription-regulating events [39]. Hence, we also investigated the role of p300 in LTA-induced MMP-9 expression. Pretreatment with the p300 inhibitor garcinol (GR343, 0.1-10 μM) concentration-dependently inhibited LTA-induced MMP-9 expression (Figure 6a). To determine whether the phosphorylation of p300 is essential for LTA-induced MMP-9 expression, we found that LTA stimulated phosphorylation of p300 in a time-dependent manner (Figure 6b). To determine whether PKC and Nox/ROS are involved in LTA-stimulated p300 phosphorylation, as shown in Figure 6c, pretreatment with Gö6976 (1 μM), DPI (1 μM), NAC (10 mM) or Apo (10 μM) attenuated LTA-stimulated p300 phosphorylation, indicating that LTA-stimulated p300 phosphorylation was mediated through the PKCα/Nox/ROS cascade in RBA-1 cells. To confirm the role of p300 in LTA-induced MMP-9 expression, as shown in Figure 6d, transfection with p300 siRNA knocked down the expression of p300 protein and inhibited MMP-9 induction by LTA. These results demonstrated that the PKCα/Nox/ROS-mediated p300 cascade plays an important role in LTA-induced MMP-9 expression in RBA-1 cells.

### LTA induces MMP-9 promoter activity and astrocytic migration

**Figure 7:**
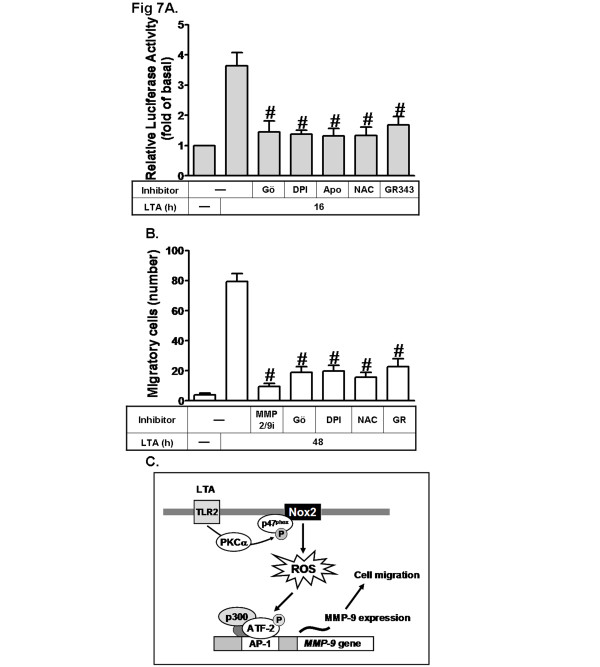
**LTA enhances MMP-9 promoter activity and astrocytic migration. a** Cells were transiently cotransfected with pGL-MMP9-Luc and pGal for 24 h, pretreated with Gö, DPI, Apo, NAC and GR343 for 1 h, and then incubated with LTA for 16 h. The promoter activity was determined. **b** RBA-1 cells were plated on coverslips and grew to confluence; the coverslips were transferred to a new 10-cm dish containing serum-free medium for 24 h. Cells were pretreated with MMP2/9i, Gö, DPI, NAC or GR343 for 1 h, and then incubated with LTA (50 μg/ml) for 48 h. Phase contrast images of RBA-1 cells were taken. The number of LTA-induced cell migrations was counted. Data are expressed as the mean ± SEM of three independent experiments. ^#^*P* < 0.01, as compared with the cells stimulated with LTA alone. **c** Scheme of the LTA-mediated signaling pathways linked to MMP-9 expression and cell migration in brain astrocytes. Action of LTA to its receptors (TLR2) results in activation of PKCα/Nox2/ROS cascade. MMP-9 transcription is ATF-2/AP-1-dependently regulated by a ROS-dependent pathway. Moreover, p300 plays the role of an assistant mediator in these responses. This signaling pathway might contribute to sustained expression of MMP-9, which is required for cell migration in RBA-1 cells.

We further investigated whether LTA-stimulated MMP-9 transcription activity was also mediated through this ROS-dependent pathway. The MMP-9 promoter was constructed into a pGL3-basic vector containing the luciferase reporter system (pGL-MMP-9-Luc), which contained AP-1 binding sites. LTA-induced MMP-9 promoter activity was attenuated by pretreatment with Gö6976 (Gö, 1 μM), DPI (1 μM), apocynin (Apo, 10 μM), NAC (10 mM) or GR343 (10 μM) (Figure 7a). To demonstrate the functional activity of MMP-9 induced by LTA, we evaluated cell migration of RBA-1 cells. The images of cell migration were taken and counted at 48 h induction by LTA (50 μg/ml). As shown in Figure 7b, LTA-induced cell migration was significantly blocked by pretreatment with the inhibitors of MMP2/9 (2/9i), PKCα (Gö, 1 μM), Nox (DPI, 1 μM), ROS (NAC, 10 mM) or p300 (GR, 10 μM). Taken together, these results suggested that upregulation of MMP-9 and its activity via the PKCα-dependent Nox/ROS and p300 pathway is required for enhancing cell migration induced by LTA in RBA-1 cells.

## Discussion

Excessive MMP-9 expression and activity are associated with sustained inflammation and BBB breakdown, leading to brain injury. Blockade of MMP-9 activity by pharmacological inhibitors or gene knockout strategies provide protective effects against cerebral ischemia [39,40]. Moreover, the gram-positive bacterium *Streptococcus pneumoniae* is the most common cause of acute bacterial meningitis worldwide [41,42], revealing a close relationship between LTA challenges and CNS diseases. The pathogenic progression involves glial activation and TLR2 signaling stimulated by LTA, which are linked to inflammatory neurodegeneration [9-11]. Additionally, LTA exhibits detrimental effects on brain cellular functions, including induction of apoptosis, production of oxidative stresses and disruption of BBB following group B *Streptococcus* or *Staphylococcus aureus* challenge in the CNS [9-11]. Despite an obviously important role of LTA in brain diseases, the processes by which LTA was implicated in astrocytic functions are not completely understood. Thus, we investigated the mechanisms underlying LTA-induced MMP-9 expression and its effects. Our recent studies have demonstrated that c-Src-dependent NF-κB and Ca^2+^/CaMKII-dependent JNK/c-Jun pathways mediate LTA-induced MMP-9 expression and cell migration in RBA-1 cells [43,44]. Here, we further demonstrated that a PKCα/Nox2/ROS-dependent ATF2/AP-1 and p300 pathway is also involved in LTA-induced MMP-9 expression and cell migration in brain astrocytes (Figure 7b). Based on these findings, Figure 7c depicts a model for the PKCα-mediated activation of p47^phox^/Nox2/ROS-dependent ATF2/AP-1 and p300 signaling pathway implicated in LTA-induced MMP-9 expression and cell migration in RBA-1 cells. On the basis of these results and our previous studies, we suggested that LTA-induced MMP-9 expression may be mediated through diverse signaling molecules and pathways, which make joint efforts to enhance MMP-9 gene expression and cell migration in brain astrocytes. However, we will further investigate the relationship among these different pathways associated with MMP-9 expression in the future.

The ROS may exert a key role in the normal physiological functions and the inflammatory responses [22,45]. Several lines of evidence have indicated that ROS play a causative role in numerous disease pathologies such as ischemia/reperfusion injury and degenerative diseases such as atherosclerosis, arthritis and neurodegeneration [24,25]. In the brain, the role of ROS extends to the control of vascular tone, which is tightly modulated by metabolic activity within neurons [21,23]. Increasing evidence also attributes the cellular damage in neurodegenerative disorders such as AD to oxidative stress that is due to the generation of free radicals implicated in brain inflammatory disorders [24,25,45]. The effects of LTA on ROS generation have been reported in human renal [27] and tracheal diseases [26]. In this study, we found and confirmed that LTA-induced MMP-9 expression is mediated through Nox-dependent ROS generation in RBA-1 cells (Figure 1). Consistently, many reports have also shown that ROS are a major signaling factor that mediates microglial activation induced by inflammatory mediators, including LPS [46]. Herein we are the first group to establish that Nox-dependent ROS generation contributes to upregulation of MMP-9 induced by LTA in brain astrocytes.

The Nox is considered to be a major source of ROS in several physiological and pathological processes [21,35]. To date, five Nox isotypes have been discovered, including Nox1-5 [34]. Several reports have shown that Nox1, Nox2 and Nox4 are expressed in brain cells [21,34], suggesting that these Nox isotypes may be essential for ROS generation. However, the role of these enzymes in the upregulation of brain astrocytic MMP-9 is still unclear. Our data demonstrated that Nox activity is involved in these responses by pretreatment with a Nox inhibitor DPI (Figures 2a-c). Moreover, we also found that Nox1, Nox2 and Nox4 are expressed in RBA-1 cells by RT-PCR (data not shown). The data of knockdown distinct Nox by transfection with their respective siRNA indicated that LTA-induced MMP-9 expression was mediated through activation of Nox2 in RBA-1 cells (Figure 2d). The data further showed that phosphorylation and translocation of the p47^phox^ subunit, a component of Nox2, is crucial for LTA-induced these responses (Figure 3). These results are consistent with previous studies showing that Nox is expressed in astrocytes, contributes to ROS generation [47,48] and leads to LPS-induced MMPs expression in Raw264.7 cells [49]. We also suggested that the LTA-stimulated p47^phox^-dependent Nox2/ROS signal and MMP-9 expression might be mediated through PKCα in RBA-1 cells (Figure 4). It is consistent with the report indicating that PKCα can phosphorylate p47^phox^, and induce both its translocation and then Nox activation in human neutrophils [36].

The excessive increase of oxidative stress during injuries can modulate the pattern of gene expression through various transcription factors. Here we focus on the roles of transcription factor AP-1, which is well known to be modulated during oxidative stress associated with inflammatory diseases [50]. Accumulating evidence has shown that MMP-9 is upregulated via an AP-1-dependent manner in various cell types [5,18]. Upon stimulation, ATF-2 is known to form heterodimers with Jun family transcription factors and bind to a specific AP-1 site in the promoter region of target genes, and enhances the gene transcription [51]. Our data showed that ATF-2 phosphorylation is essential for LTA-induced MMP-9 expression, determined by AP-1 inhibitor (TSIIA) (Figure 5). Such inhibitory effects were also confirmed by transfection with ATF-2 siRNA on MMP-9 expression (Figure 5a). Theses results are consistent with the mechanisms of MMP-2 expression in human breast epithelial cells [52]. Moreover, AP-1 binding sites have been identified in the MMP-9 gene promoter [3], which might explain the modulation exerted by LTA. Our data further showed that LTA enhanced phospho-ATF-2 binding to MMP-9 promoter, determined by a ChIP-PCR assay (Figure 5d), consistent with the responses of phosphorylation of ATF-2 (Figure 5b). These results suggested that LTA stimulated ATF-2/AP-1 phosphorylation and binding activity via a ROS-dependent pathway, consistent with ROS-dependent activation of ATF-2 being involved in H9c2 cells [53].

Moreover, the transcription co-activator p300 is vital for the co-activation of several transcription factors such as AP-1 in the transcription machinery, which has a significant role in the activation of AP-1-mediated gene expression for proinflammatory mediators [39]. Our results indicated that p300 plays an important role in LTA-induced MMP-9 expression (Figure 6a). Moreover, we found that LTA increased phosphorylation of p300 is also mediated through a PKC(α)/Nox(2)/ROS cascade (Figures 1b and c). The result of p300 siRNA transfection further confirmed that p300 may contribute to enhance transcriptional activation of MMP-9 by LTA (Figure 6d). It was consistent with previous studies indicating p300-dependent MMP-9 expression by BK or IL-1β in astrocytes [17,54] and a recent study showing that ROS-dependent p300 activation leads to cPLA_2_ expression by cigarette smoke extract in human tracheal smooth muscle cells [55]. Subsequently, we also demonstrated that LTA-stimulated MMP-9 transcriptional activity via the same pathway by a wild-type rat MMP-9 promoter-luciferase reporter plasmid (pGL-MMP-9-Luc) construct (Figure 7a). These results indicated that LTA-induced MMP-9 expression is mediated through a PKC(α)-dependent Nox(2)/ROS signal, associated with activation of transcription factor ATF-2/AP-1 in RBA-1 cells. Consistently, in rat brain primary cultured astrocytes, we confirmed that LTA-induced MMP-9 expression may mediate through the same identified signaling (Figure 1d).

Cell motility is a fundamental process during embryonic development, wound healing, inflammatory responses and tumor metastasis [56]. It has been reported that MAPKs, NF-κB, AP-1 and MMP-9 [17,18] contribute to cell motility in different cell types. In the study, we further demonstrated that in brain astrocytes, activation of a Nox-dependent ROS signal linking to ATF-2/AP-1 and p300, mediated through activation of PKCα, is essential for LTA-induced MMP-9 gene expression and cell migration.

## Conclusions

In summary, these results demonstrated that LTA induces MMP-9 expression via sequential activation of PKCα, p47^phox^/Nox2, ROS, and transcription factor ATF2/AP-1 and co-activator p300, leading to the promotion of astrocytic migration. Although the molecular basis of the Nox-mediated redox signal in immune, cardiac and renal diseases is well established, the role of the Nox-mediated redox signal during acute or chronic brain inflammation is still unclear. We demonstrated that the Nox-mediated ROS signal may be a pivot molecule linking the PKCα and AP-1 signal; this leads to expression of MMP-9 in CNS infection. Pharmacological approaches suggest that targeting MMP-9 and its specific upstream signaling components should yield useful therapeutic targets for CNS inflammatory diseases upon infection with gram-positive bacteria.

## Competing interests

The authors declare that they have no competing interests.

## Authors’ contributions

CCL, RHS and LDH designed and performed experiments, acquisition and analysis of data, and drafted the manuscript. HLH and CMY co-conceived the study, participated in its design and coordination, were involved in drafting the manuscript and revising it critically for important intellectual content, and gave final approval of the version to be published. All authors read and approved the final version of this manuscript.
